# Supplementary material for: Characterizing acceptable and appropriate implementation strategies of a biobehavioral survey among men who have sex with men and others assigned male who have sex with men in Zimbabwe
Source: PLOS Glob Public Health. 2022 Oct 26;2(10):e0001097. doi: 10.1371/journal.pgph.0001097 (PMC10021218; doi:10.1371/journal.pgph.0001097)
Supplement: S1 Text — (PDF) [file pgph.0001097.s001.pdf]

Appendix 9: INTERVIEW GUIDE FOR MSM

**Date**	**|___||___| / |___||___| / |___||___|** *(dd/mm/yyyy)*
**Primary Interviewer Name**	
**Note taker name(s)**	
**Venue**	
**Start Time**	**|___||___| : |___||___|** *(hour/min)*
**End Time**	**|___||___| : |___||___|** *(hour/min)*
**How was this participant referred to be interviewed?**	
**Name of electronic file**	

NB: The text to be read is in normal font and the probing questions and instructions are in italics. Not all the probing questions need to be asked if the discussion flows freely

Introduction

Before I start the interview, I kindly ask you turn off your cell phone and other mobile devices. I will be asking you questions about MSM in your city.

We are conducting this survey with men who have sex with men (MSM) in Harare and Bulawayo to learn about their risks for HIV and other STIs. What we learn from this survey will help us make suggestions for how to improve delivery of health services for MSM in Zimbabwe.

We are asking for your ideas and opinions that can help us better understand risks for HIV and sexually transmitted infections (STI) among MSM in Zimbabwe. We will ask you some questions about MSM, about how they socialize, what kinds of different groups of MSM there are, and what ways MSM can be made aware of health services and information related to prevention and treatment of HIV and STIs. We will also ask you some questions about how to improve health services for MSM and about MSM experiences using these services. Towards the end of the interview we will provide you with a blank map of [name of the survey area] or with its main features (e.g., river, bridges) and ask you to note the locations where MSM gather. We will call these places “hotpots”. We will ask you to estimate the average number of MSM expected to be found in each hotspot and characterize the types of social groups in each location who may be able to help promote HIV/STI outreach efforts to MSM. The hotspot map will only be used by the survey team and will not be shared outside of the survey team.

During the interview, we ask that you not use real names or anything that would identify others. However, please be sincere about your opinions and experiences as this will help us make recommendations that are feasible and will help meet the needs of these populations. Additionally, when I say “friends”, “colleagues”, “peers” or “people like you”, I mean people you know who are MSM, or gay. MSM may not identify as gay but they have sex with men.

Do you have any questions before we start? *(Take time to address all questions and concerns)*

1. GENERAL INFORMATION/TRENDS

Let’s start by talking a bit about men who have sex with men in [name of the survey area].

1.1. How would you describe in general the population of MSM in [name of the survey area]?

1.2. Could you tell me about how the MSM that you know interact with each other?

a. *What kinds of social or other activities do they do together?*
b. *Which bars, restaurants or similar places do you and your friends go to? Are these places frequented mainly by MSM or are they ‘mixed’?*
c. *Where in the city do the activities for MSMs take place?*
d. *How often and under what conditions do the MSM you know see each other? (eg, work, social activities, social organizations)*
e. *How often and under what conditions do you interact with MSM who are from other parts of the city?*
f. *Do they move a lot in and out of [name of the survey area]? Do a lot of MSM move to [name of the survey area] from the regions. If so, why?*
g. *Besides [name of the survey area], where are some MSM you know from?*
h. *Besides Zimbabwe, from which countries are some MSM you know from? (if so specify Africa, outside Africa)*
i. *How often do MSM you know go to other countries?*
j. *What other MSM do you know who are different from you?*

1.2 How old are most of your peers? Do MSM tend to associate based on age? What other factors might MSM social groups form around? Do you know MSM of different ages?

1.3 How large do you think the population of MSM is in [name of the survey area]?

a. *How many MSM do you think there are? What percentage of men do you think are MSM?*

1.4 Have you noticed any changes or trends over the past year with regard to MSM? (eg, new populations/groups, new hangouts, new or changing risk behaviors)

1.5 What are the different kinds of types or social groups of men who have sex with men?

a. *How would you describe them?*
b. *How much contact is there between these groups?*
c. *How do these different groups interact when working?*
d. *How do they interact socially?*
e. *What suggestions do you have for reaching different sub-groups?*

1.6 What proportion of the men who have sex with men whom you know are married? How many have girlfriends or also have sex with women?

a. *Most, some, few?*

1.7 Of the MSM you know, about how many are openly gay/open about their sexuality with family and non-gay friends?
   a. *Would you say most of them, some or very few?*
   b. *What proportion of the MSM you know are gay-identified?*

1.8 How many MSM do you know? Please take your time to think about this. Don't tell me their names; I just want to know about how many people you know
   a. *How many live in [name of the survey area]?*
   b. *How many have you seen in the past <u>three</u> months*
   c. *How many of these people have you seen in the past month?*
   d. *How many MSM are in your mobile phone contact list?*

2 HEALTH OUTREACH

<u>*Location*</u>

2.1. Where would you and your peers feel comfortable coming to seek healthcare or talk to someone about their own health? *What might be some convenient and safe locations?*
   a. *Clinic? NGO? Other location?*
   b. *Would a home visit by healthcare staff be acceptable?*
   c. *Would it be quiet and private?*
   d. *What are the areas that we should avoid?*
   e. *What are the areas you personally would not go to?*
   f. *What about certain areas that specific type of men would not feel comfortable going to certain areas e.g. non-homosexual identified, men from other areas?*

2.2 What would make a health care facility most comfortable for other MSM?

2.3 What are the most convenient times of the day for MSM to go to receive health information and/or seek attention for their own health issues? Morning (8am-12pm)? Afternoon (12pm-5pm)? Evening (5pm-10pm)?

2.4 What days of the week do you think MSM would be most likely to go seek attention for health issues?
   a. *What about the weekend?*

<u>*Languages*</u>

2.5 What languages do you speak? What is your main language? What do most of your peers speak? How many of your friends would speak neither English, Shona, and Ndebele?

2.6 What languages can you read? Can most of your peers read? Are there MSM who are not able to read? How would you describe these men?
   a. *Is there a difference between the MSM who are literate and those who are not? eg greater tendency among literate to self-identify as gay. Do they socialize together?*
   b. *How can we publicize information about health issues that may affect t MSM to MSM who are illiterate?*

<u>*Staffing*</u>

2.7 Describe the type of person with whom you would feel most comfortable answering personal questions about your health.

a. *Would men and women both be acceptable as interviewers?*
b. *Are there people you would <u>not</u> be comfortable with?*

2.8 Describe the type of person with whom you would feel most comfortable taking rectal swabs and testing you for HIV?
a. *Would men and women both be acceptable?*
b. *Are there people you would <u>not</u> be comfortable with?*

3 STIGMA AND SERVICE PROVISION

3.1 What are the support organizations that are well known among you and your peers? Are you part of any group or organization for gay men?
a. *What is the name of the organization (formal or informal)?*
b. *Is it exclusively for gay men/MSM?*
c. *How does it provide support?*

3.2 Can you give me some concrete examples where someone was stigmatized, humiliated or insulted because of their MSM activity? (E.g. verbal insults, loss of house, job, etc.)
a. *If so by whom?*
b. *For what reason?*
c. *How often does this happen?*

3.3 Can you give me some concrete examples where someone was subject to physical violence because of their MSM activity? (E.g. verbal insults, loss of house, job, etc.)
a. *If so by whom?*
b. *For what reason?*
c. *How often does this happen?*

3.4 If an MSM is subjected to stigmatization or violence, who can they turn to? Who can help them? Are there some people/groups who protect MSM from insults and violence?
a. *Probe to see who these people are and what they do to protect MSM?*
b. *Who can they not turn to (for example, what is the reaction of the police?)*
c. *Can you give me some concrete examples?*

3.5 What kinds of HIV or STI prevention services do MSM use?

3.6 How are MSM received when they seek treatment at formal (government) health services?
a. *What factors/attitudes among health care workers could make MSM reluctant to use government health services?*

4. SURVEY PARTICIPATION

As you know, we are looking for advice for our future survey. In the future survey we will interview people about HIV and what they do to prevent it. We will ask questions about how they meet people and about things they do that put them at risk for HIV. We also want to give free HIV testing and counselling. We will give back the test results to the people tested in the survey. If they test positive for HIV, we will tell them where they can get care and treatment. People will spend up to two hours in the future survey.

4.1 What are your initial thoughts about this survey?
a. *How would you feel about joining this survey?*
b. *What would keep you from joining this survey?*
c. *What would make you feel more comfortable about joining?*

4.2 Do you think other MSMs would be willing to join in that future survey? Why, or why not?
  a. *What would make your friends feel more comfortable about joining?*
  b. *How would you encourage a friend to join the survey? Especially one who is reluctant?*

4.3 Would you or your peers be willing to talk openly with an interviewer about personal sexual behavior if it is anonymous and in a private setting? What about your drug use?

4.4 What do we need to know to make the survey a success?
  a. *How can we let people know about the survey?*
  b. *What can we do to get a lot of people to join the survey?*
  c. *. Who are the key people or groups we should talk to, to gain support for the survey?*

5. RDS: COUPON DESIGN AND RECRUITMENT

Let me describe the method we want to use to find people to join the survey. We would give participants three coupons to give out to peers who are also MSMs. For each friend who shows up to the survey location with the coupon and participates, the person who referred them will get a small amount of money.

Your friend would also be interviewed, get free HIV testing and STI screening and treatment, be told about HIV and how to prevent it, and get coupons to give out to his friends so they can also participate. Now we would like to know:

5.1 How would you feel about giving a coupon to your peers and asking them to do the survey?
  a. *Do you think these people would agree to join in the survey?*
  b. *b Do you think these people would be willing to refer others to participate?*

5.2 Do you know of any especially influential MSMs who may be good at referring people? This would be someone who knows a lot of other MSMs and is well liked by peers.
  a. *Would they be willing to talk to us?*
  b. *Can you tell us a little bit about them? [Ask participants to ask identified peer leaders to contact survey coordinator. Give them referral card with contact information for survey coordinator.]*

5.3 What color should the coupon be?

5.4 What information should on the coupon? Survey hours, contact phone number, survey location, survey name?
  a. *Can you think of any information that really needs to be there to make it easy for participants to come see us?*
  b. *Can you think of anything that if on the coupon would make you not want to join?*
  c. *How could we adapt the coupon for those who can't read?*

6. MAPPING

Now I'm going to blank map of [name of the survey area] with its main features (e.g., river, bridges). Each map is marked with your survey ID. On the map, please note the following:

- Locations where MSM congregate (these areas will be considered as MSM hotpots)
- Your estimate of the average number of MSM expected to be found in each hotspot at a given time

Please complete this exercise first on your own and then we will discuss. I will then create a table that notes the name of each hotspot, the number it was assigned on the map, the day and time when the maximum and minimum number of MSM can be found, and the type and scale of risk in each location (e.g., presence of commercial MSM sex workers, presence and characteristics of facilitators who may help in promoting HIV/STI outreach efforts)

That is the end of our interview.

Thank you so much for sharing your thoughts with me.

Do you have any questions, or is there anything that you would like to add before we end?

If you have further thoughts about any of the issues we discussed today, please call *[INSERT NUMBER WHERE INTERVIEWER CAN BE CONTACTED]*

TO BE COMPLETED BY THE INTERVIEWER:

Please note your impressions about the session, its main themes and the comments and reactions of participants

INTERVIEW WAS: ____ ROUTINE ____ NOT ROUTINE

IF NOT ROUTINE, WHY:

ANY ADVERSE REACTIONS IN THE INTERVIEW: ____ YES ____ NO

IF YES, SPECIFY:

OTHER OBSERVATIONS/COMMENTS:
