# Supplementary material for: Characterizing acceptable and appropriate implementation strategies of a biobehavioral survey among men who have sex with men and others assigned male who have sex with men in Zimbabwe
Source: PLOS Glob Public Health. 2022 Oct 26;2(10):e0001097. doi: 10.1371/journal.pgph.0001097 (PMC10021218; doi:10.1371/journal.pgph.0001097)
Supplement: S2 Text — (PDF) [file pgph.0001097.s002.pdf]

Appendix 7: FOCUS GROUP GUIDE

**Date**	**|___||___| / |___||___| / |___||___|** *(dd/mm/yyyy)*
**Moderator Name**	
**Notetaker Name**	
**Venue**	
**Start Time**	**|___||___| : |___||___|** *(hour/min)*
**End Time**	**|___||___| : |___||___|** *(hour/min)*
**# of Participants at start of Focus Group**	**|___||___|**
**How many participants remained full-time?**	**|___||___|**
**Name of electronic file**	**FGD_City_DDMMYY_##**

NB: The text to be read is in normal font and the probing questions and instructions are in italics. Not all the probing questions need to be asked if the discussion flows freely.

Introduction
Before I start the Focus Group Discussion, I will kindly ask you to turn off your cell phone and other mobile devices. I will be asking you questions about men who have sex with other men. We should respect what others say and not speak while others are speaking. After the focus group, do not share with others what people in the group say today.

We are conducting this survey with men who have sex with men (MSM) in Harare and Bulawayo to learn about their risks for HIV and other STIs. What we learn from this survey will help us make suggestions for how to improve delivery of health services for MSM in Zimbabwe.

We are asking for your ideas and opinions that can help us better understand risks for HIV and sexually transmitted infections (STI) among MSM in Zimbabwe. We will ask you some questions about MSM, about how they socialize, what kinds of different groups of MSM there are, and what ways MSM can be made aware of health services and information related to prevention and treatment of HIV and STIs. We will also ask you some questions about how to improve health services for MSM and any information about what it is like to use these services. Towards the end of the Focus Group Discussion, we will provide you with a blank map of [name of the survey area] or with its main features (e.g., river, bridges) and ask you to note the locations where MSM gather. We will call these places “hotpots”. We will ask you to estimate the average number of MSM expected to be found in each hotspot and characterize the types of social groups in each location who may be able to help promote HIV/STI outreach efforts to MSM. The hotspot map will only be used by the survey team and will not be shared outside of the survey team.

During the Focus Group Discussion, we ask that you not use real names or anything that would identify others. However, we request for your honest opinions as this will help us make

recommendations that are feasible and will help meet the needs of MSM. We ask each participant to keep what is said in this discussion to themselves and not go about gossiping about other people's contributions. Additionally, when I say "friends", "colleagues", "peers" or "people like you", I mean people you know who have sex with other men. Some MSM may not identify as homosexual, gay, or MSM but they may have sex with men.

Do you have any questions before we start? *(Take time to address all questions and concerns)*

1. GENERAL INFORMATION/TRENDS

Let's start by talking a bit about men who have sex with men in [name of the survey area]?

1.1. How would you describe in general the population of MSM in [name of the survey area]?

1.2. Could you tell me about how the MSM that you know interact with each other?
- a. *What kinds of social activities do they do together?*
- b. *Which bars, restaurants or other places do MSMs go to? Are these place frequented mainly by MSM or are they 'mixed' with other non-MSM individuals?*
- c. *Where in the city do the activities take place?*
- d. *How often and under what conditions do the MSM you know see each other? (e.g. work, social activities, social organizations)*
- e. *How often and under what conditions do you interact with MSM who are from other parts of the city?*
- f. *Do they move in and out of Harare/Bulawayo a lot? Do a lot of MSM move to Harare/Bulawayo from the regions? If so, why? Are some MSM you know from other countries (if so specify Africa, outside Africa)?*
- *g.* What age group do you think most MSMs are in? Do young MSM primarily hang out with young MSM?

1.3. How large do you think the population of MSM is in [name of the survey area]?
- a. *How many MSM do you think there are in* [name of the survey area]*?*
- b. *What percentage of all men in* [name of the survey area].*do you think are MSM?*

1.4. Have you noticed any changes or trends over the past year with regard to MSM? (e.g. new populations/groups, new hangouts, new or changing risk behaviors)

1.5. What are the different kinds of types of MSM social groups that are important to know about for health providers and people who provide services to MSM?
- a. *How would you describe them?*
- b. *How much contact is there between these groups?*
- c. *How do these different groups interact when working?*
- d. *How do they interact socially?*
- e. *What suggestions do you have for reaching different sub-groups?*

1.6. What proportion of MSM would you say are married? How many have girlfriends or also have sex with women?
- a. *Most, some, few?*

2. HEALTH OUTREACH

Location

2.1. Where would MSM feel comfortable coming to seek healthcare or talk to someone about their own health? *What might be some convenient and safe locations?*
   a. *Clinic? NGO? Other location?*
   b. *Would a home visit by healthcare staff be acceptable?*
   c. *Would it be quiet and private?*
   d. *What are the areas that we should avoid?*
   e. *What are the areas you personally would not go to?*
   f. *What about certain areas that specific type of men would not feel comfortable going to certain areas e.g. non-homosexual identified, men from other areas?*

2.2. What are the most convenient times of the day for MSM to go to receive health information and/or seek attention for their own health issues? Morning (8am-12pm)? Afternoon (12pm-5pm)? Evening (5pm-10pm)?
   a. *How do you think the most convenient hours differ by kind of MSM? (eg, older/younger, open/private about being MSM)*

2.3. What days of the week do you think MSM would be most likely to go to seek attention for health issues?
   a. *What about the weekend?*
   b. *How do you think the most convenient days of the week differ by kind of MSM? (eg, older/younger, open/private about being MSM)*

Staffing

2.4. How would you describe the type of person with whom MSMs would feel most comfortable answering personal questions about their health?
   a. *Would men and women both be acceptable as interviewers?*
   b. *What about homosexual men? Straight men?*
   c. *Who would MSM not be comfortable with?*

3. STIGMA AND SERVICE PROVISION

3.1. What are the support organizations that are well known among MSMs?
   a. *What is the name of the organization (formal or informal)?*
   b. *What type of support do they provide? Probe for health support, legal/human rights, etc.*
   c. *Is it exclusively for MSM?*
   d. *How does it provide support?*

3.2. How are MSM treated/viewed in Zimbabwe?

3.3. If stigmatized, what kind of stigmatization, humiliation or insults do MSM face?
   a. *Mostly by whom?*
   b. *Mostly for what reason?*
   c. *How common is this?*

3.4. What kind of physical or sexual violence do MSM face?
   a. *Mostly, by whom?*
   b. *Mostly, for what reason?*
   c. *How common is this?*

3.5. If an MSM is subjected to stigmatization or violence, who can they turn to? Who can help them? Are there some people/groups who protect MSM from insults and violence?
   a. *Who are these people and what they do to protect MSM?*
   b. *Who can they not turn to (for example, what is the reaction of the police?)*

c. *Can you give me some concrete examples?*

3.6. What kinds of HIV or STI prevention services do MSM use?
a. How can these services be improved?
b. What services do they need?

3.7. How are MSM received when they seek treatment at formal (government) health services?
a. *What factors/attitudes among health care workers could make MSM reluctant to use government health services?*
b. *Are there health care providers who are okay serving MSM? Who are they?*

4. SURVEY PARTICIPATION

I will now move on to ask you questions about participation in this survey

4.1 What are your initial thoughts about this survey?
a. *How would you feel about joining this survey?*
b. *What would keep you from joining this survey?*
c. *What would make you feel more comfortable about joining?*

4.2 Do you think other MSMs would be willing to join in that future survey? Why, or why not?
a. *What would make your friends feel more comfortable about joining?*
b. *How would you encourage a friend to join the survey? Especially one who is reluctant?*

4.3 Would you or your peers be willing to talk openly with an interviewer about personal sexual behavior if it is anonymous and in a private setting? What about your drug use?

4.4 What do we need to know to make the survey a success?
a. *How can we let people know about the survey?*
b. *What can we do to get a lot of people to join the survey?*
c. *. Who are the key people or groups we should talk to, to gain support for the survey?*

5. BIOLOGICAL TESTING

5.1 How do you feel about taking a free HIV test as part of this survey?
a. *How do you feel about giving blood from a vein in your arm or finger prick?*
b. *Would free HIV testing make people more or less likely to join the survey? Why?*
c. *How can we make HIV testing more acceptable?*

5.2 Which kind of person would you prefer to take swabs and test you for HIV?
a. *Would men and women both be okay?*
b. *Are there people you would not be comfortable with?*

6. INTERVIEW ADMINISTRATION

Now I have some questions about how to collect personal information in the next survey.

6.1 Would you feel comfortable being honest with an interviewer? Would you be more comfortable being honest to a computer or phone?
6.2 How would you feel about a trained researcher asking you the interview questions?
6.3 Describe the type of person with whom you would feel ok to answer personal questions in an interview.

7. SURVEY LOGISTICS

7.1 For the future HIV survey, would you like us to come to the places you socialize to do the survey?
7.2 Would you want to be invited to a specific private location like a clinic, private office/building, or other space where you can do the survey when you want?

8. RDS: Compensation

We will want to give something to participants for their time and transport. We want to make sure we do not give too much to avoid people pretending that they are a MSMs. We also do not want to give something so big that people will join the survey just to get it.

8.1 How do you think we can best compensate people for their time and transport?
   a. *Would a gift (i.e., phone credit/airtime, food voucher) be acceptable?*
   b. *Would money be acceptable?*
   c. *How much would it cost to get to the survey site?*
   d. *How much should we give for time spent doing the survey?*

8.2 Would health screenings make people more likely to join?
We will ask participants to return to the survey office for a second visit about two weeks after the first. This second visit will take about 30 minutes. During the visit, participants will get their test results and will be given compensation for each of their recruits who participated.
8.3 Would you and your peers be able to return for a second visit after two weeks? Why/why not?
8.4 We would like to compensate participants who recruit peers. What do you think we should give participants for recruiting peers?

9. RDS: Location, Days, Times, Etc.

9.1 What kind of place would you like to see as the main survey site location? What might be some convenient and safe locations?
9.2 Where would you and your peers feel comfortable coming to participate in a survey? Rented apartment or office? Clinic? NGO? Other location?
9.3 How easy is it for people to get there? How do you think you and your peers would travel to the survey site?
   a. *Probe for public transit, shared taxi, private taxi, private motorcycle, etc.*
9.4 About how much would you expect transport to the interview site to cost for most people? Probe to see if this cost is for public transit, shared taxi, private taxi, private motorcycle etc.
9.5 What areas of town are unsafe and should be avoided for the survey?
9.6 What types of MSMs would not feel comfortable going to certain areas?
9.7 Are there any locations where you would not want to be surveyed?
9.8 What would make a survey site most comfortable for other MSMs?
9.9 What times of the day are best for MSMs to go to an interview site to take part in a survey?
   a. *Morning (8 AM–12 PM)?*
   b. *Afternoon (12 PM–5 PM)?*
   c. *Evening (5 PM–10 PM)?*
9.10 What days of the week do you think MSMs are best for people to join the survey? What about the weekend?
9.11 Would appointments or open-walk in times work better?
9.12 We may also ask other MSMs to participate in a similar study at the same time. How would you feel about being at the same survey site as they?

10. RDS: Coupon Design and Recruitment

Let me describe the method we want to use to find people to join the survey. We would give participants three coupons to give out to peers who are also MSMs. For each friend who shows up to the study location with the coupon and participates, the person who referred them will get a small amount of money.

Your friend would also be interviewed, get free HIV testing and STI screening and treatment, be told about HIV and how to prevent it, and get coupons to give out to his friends so they can also participate. Now we would like to know:

10.1 How would you feel about giving a coupon to your peers and asking them to do the survey?
   a. *Do you think these people would agree to join in the study?*
   b. *Do you think these people would be willing to refer others to participate?*

10.2 Do you know of any especially influential MSMs who may be good at referring people? This would be someone who knows a lot of other MSMs and is well liked by peers.
   a. *Would they be willing to talk to us?*
   b. *Can you tell us a little bit about them? [Ask participants to ask identified peer leaders to contact survey coordinator. Give them referral card with contact information for survey coordinator.]*

10.3 What color should the coupon be?

10.4 What information should on the coupon? Survey hours, contact phone number, survey location, survey name?
   a. *Can you think of any information that really needs to be there to make it easy for participants to come see us?*
   b. *Can you think of anything that if on the coupon would make you not want to join?*
   c. *How could we adapt the coupon for those who can't read?*

11. MAPPING

Now I'm going to blank map of this city with its main features (e.g., river, bridges). Each map is marked with your survey ID. On the map, please note the following:

- Locations where MSM congregate (these areas will be considered as MSM hotpots)
- Your estimate of the average number of MSM expected to be found in each hotspot at a given time

Please complete this exercise first on your own and then we will discuss as a group. I will then create a table that notes the name of each hotspot, the number it was assigned on the map, the day and time when the maximum and minimum number of MSM can be found, and the type and scale of risk in each location (e.g., presence of commercial MSM sex workers, presence and characteristics of facilitators who may help in promoting HIV/STI outreach efforts)

That is the end of the discussion.

Thank you so much for sharing your thoughts with me.

Do you have any questions, or is there anything that you would like to add before we end?

If you have further thoughts about any of the issues we discussed today, please call *[INSERT NUMBER WHERE MODERATOR CAN BE CONTACTED]*

TO BE COMPLETED BY THE MODERATOR:

Please note your impressions about the session, its main themes and the comments and reactions of participants

FOCUS GROUP WAS: ____ ROUTINE ____ NOT ROUTINE

IF NOT ROUTINE, WHY:

ANY ADVERSE REACTIONS IN THE FOCUS GROUP : ____ YES ____ NO

IF YES, SPECIFY:

OTHER OBSERVATIONS/COMMENTS:

TO BE COMPLETED BY THE NOTETAKER(S):

Please note your impressions about the session, its main themes and the comments and reactions of participants

FOCUS GROUP WAS: ____ ROUTINE ____ NOT ROUTINE

IF NOT ROUTINE, WHY:

ANY ADVERSE REACTIONS IN THE FOCUS GROUP : ____ YES ____ NO

IF YES, SPECIFY:

OTHER OBSERVATIONS/COMMENTS:
