# Supplementary material for: Characterizing acceptable and appropriate implementation strategies of a biobehavioral survey among men who have sex with men and others assigned male who have sex with men in Zimbabwe
Source: PLOS Glob Public Health. 2022 Oct 26;2(10):e0001097. doi: 10.1371/journal.pgph.0001097 (PMC10021218; doi:10.1371/journal.pgph.0001097)
Supplement: S3 Text — (PDF) [file pgph.0001097.s003.pdf]

Appendix 8: INTERVIEW GUIDE FOR FACILITATORS

**Date**	**|___||___| / |___||___| / |___||___|** *(dd/mm/yyyy)*
**Primary Interviewer Name**	
**Note taker name(s)**	
**Venue**	
**Start Time**	**|___||___| : |___||___|** *(hour/min)*
**End Time**	**|___||___| : |___||___|** *(hour/min)*
**Participant's association with the target population**	
**How was this participant referred to be interviewed?**	
**Name of electronic audio file**	

NB: The text to be read is in normal font and the probing questions and instructions are in italics. Not all the probing questions need to be asked if the discussion flows freely

Introduction

Before I start the interview, I kindly request that you turn off your cell phone and other mobile devices.

We are conducting this survey with men who have sex with men (MSM) in Harare and Bulawayoto learn about their risks for HIV and other STIs. What we learn from this will help us make suggestions for how to improve delivery of health services for MSM in Zimbabwe.

We are asking for your ideas and opinions that can help us better understand risks for HIV and sexually transmitted infections (STI) among MSM in Zimbabwe. We will ask you some questions about MSM, about how they socialize, what kinds of different groups of MSM there are, and what ways MSM can be made aware of health services and information related to prevention and treatment of HIV and STIs. We will also ask you some questions about how to improve health services for MSM. Towards the end of the interview we will provide you with a blank map of this city or with its main features (e.g., river, bridges) and ask you to note the locations where MSM gather. We will call these places "hotpots". We will ask you to estimate the average number of MSM expected to be found in each hotspot and characterize the types of social groups in each location who may be able to help promote HIV/STI outreach efforts to MSM.

We ask that you not use real names or anything that would identify MSM. However, please be honest about your opinions and experiences as this will help us make recommendations that are feasible and will help meet the needs of these populations.

Do you have any questions before we start? *(Take time to address all questions and concerns)*

1. BACKGROUND QUESTIONS

1.1. Can you describe your relationship with the MSM you encounter?
- *a. How have you come to know MSM?*
- *b. When you come into contact with an MSM, how do you know they are MSM?*
- *c. How do you interact with them?*
- *d. What do you do together? (eg, socialize together, work together)*

1.2. What are your feelings about MSM in general?

1.3. Regarding the MSM you know, where do you see them or meet with them?
- *a. Where do they congregate?*
- *b. In what kind of places?*
- *c. Bars, clubs, at ceremonies , etc.*

1.4. How often do you interact with MSM?
- *a. With what frequency do you see them? Do you see them regularly (every day, week, month or less frequently?*
- *b. If you do not see them regularly, why is this?*
- *c. What are there times when you see them more regularly?*

1.5. What do you do to support MSM?
- *a. Counsel them? If so, specify area*
- *b. Have someone ever talked to you about hiding their sexuality, e.g. from wives and girlfriends? Could you describe one or two of these encounters?*
- *c. Please give a concrete example (without naming names) of when you provided support.*

1.6. What do you do to protect MSM? *(eg, from violence, insults or stigmatization)?*
a. Financial support? Legal advice? Housing shelters? Mental health counseling?
b. Please give a concrete example (without naming names of when you provided protection.

1.7. How do people generally view your association with MSM?
- *a. If people criticize you, describe who these people are and what they say.*
- *b. If people support you, describe who these people are and what they say.*

1.8. Can you describe the different MSM informal groups in this city?
- *a. What names do they go by?*
- *b. What groups do you have contact with?*

c. *Of the groups you can think of, what are the groups you don't have any contact with? (eg, Male sex workers, Men who have sex with men, but do not identify themselves as 'gay', older MSM, MSM with more money or a different educational status)*

1.9. Can you describe the difficult parts of your peer outreach and counseling activities?
1.10. How have you overcome these in the past?
1.11. What has worked well in your activities?
1.12. What else could be done to improve your roles?

2.0 ACCEPTABILITY OF SURVEY/ MSM PARTICIPATION

I will now move on to ask question about the survey acceptability and participation of MSMs in the survey

2.1. How would you think MSMs feel about participating in this survey?

2.2. What would discourage them from participating in this survey?

2.3. What would make them feel more comfortable about participating?

2.4. **Only for clinicians:** We plan to offer free testing and treatment for HIV and sexually transmitted infections as part of the study. The tests involve collecting some blood. What challenges have you experienced collecting blood from people in general and specifically from MSMs?

(Probe for fear of learning they have HIV, fear of lack of confidentiality, infecting others, use in "witchcraft", etc.)

- *What would make participants feel more comfortable providing specimens?*

2.5. Would people feel more comfortable with a male or female nurse?

2.6. We will offer HIV and STI testing and referral. What other services would MSMs benefit from in your view?

(Probe to see what services are currently lacking or how existing services could be improved (medical, psychosocial, etc))

2.7. We want this survey to be helpful to MSMs. We also want to make it safe for people to join. What can we do to keep people safe?

2.8. Should we tell the police or other law enforcement agencies about the survey so they don't bother people trying to join? Or is it better not to inform them?

- *What about others (religious, military, local level government, etc.)?*

2.9. How do you think MSMs will feel about joining the survey?

- *What can we do to make it easier for such MSMs to join the survey*
- *Are there any groups or individuals we can mobilize to make it easier for MSMs to participate?*

2.10. What type of people do you know who are influential among MSMs?

- *Peer leaders? Gatekeepers? (This person need not be MSM. This would be someone who knows a lot of other MSMs and is well liked by peers.*
- *Would they be willing to talk to us?)*

That is the end of our interview.

Thank you so much for sharing your thoughts with me.

Do you have any questions, or is there anything that you would like to add before we end?

If you have further thoughts about any of the issues we discussed today, please call *[INSERT NUMBER WHERE INTERVIEWER CAN BE CONTACTED]*

TO BE COMPLETED BY THE INTERVIEWER:

Please note your impressions about the session, its main themes and the comments and reactions of participants

INTERVIEW WAS: ____ ROUTINE ____ NOT ROUTINE

IF NOT ROUTINE, WHY:

ANY ADVERSE REACTIONS IN THE INTERVIEW: ____ YES ____ NO

IF YES, SPECIFY:

OTHER OBSERVATIONS/COMMENTS:
