# Supplementary material for: Characterizing acceptable and appropriate implementation strategies of a biobehavioral survey among men who have sex with men and others assigned male who have sex with men in Zimbabwe
Source: PLOS Glob Public Health. 2022 Oct 26;2(10):e0001097. doi: 10.1371/journal.pgph.0001097 (PMC10021218; doi:10.1371/journal.pgph.0001097)
Supplement: S4 Text — (PDF) [file pgph.0001097.s004.pdf]

Appendix 10: INTERVIEW GUIDE FOR SERVICE PROVIDERS

**Date**	**|___||___| / |___||___| / |___||___|** *(dd/mm/yyyy)*
**Primary Interviewer Name**	
**Note taker name(s)**	
**Venue**	
**Start Time**	**|___||___| : |___||___|** *(hour/min)*
**End Time**	**|___||___| : |___||___|** *(hour/min)*
**Health facility or organization where s/he works**	
**How was this participant referred to be interviewed?**	
**Name of electronic audio file**	

NB: The text to be read is in normal font and the probing questions and instructions are in italics. Not all the probing questions need to be asked it ends up being answered already because of free-flowing conversation

Introduction

Before I start the interview, I kindly request that you turn off your cell phone and other mobile devices.

We are conducting this survey with men who have sex with men (MSM) in Harare and Bulawayo to learn about their risks for HIV and other STIs. What we learn from this survey will help us make suggestions for how to improve delivery of health services for MSM in Zimbabwe.

We are asking for your ideas and opinions that can help us better understand risks for HIV and sexually transmitted infections (STI) among MSM in Zimbabwe.

We will ask you some questions about MSM, about how they socialize, what kinds of different groups of MSM there are, and what ways MSM can be made aware of health services and information related to prevention and treatment of HIV and STIs. We will also ask you some questions about how to improve health services for MSM and about your experience using these services. Towards the end of the interview we will provide you with a blank map of [name of the survey area] or with its main features (e.g., river, bridges) and ask you to note the locations where MSM gather. We will call these places “hotpots”. We will ask you to estimate the average number

of MSM expected to be found in each hotspot and characterize the types of social groups in each location who may be able to help promote HIV/STI outreach efforts to MSM.

We ask that you not use real names or anything that would identify MSM. However, please be honest about your opinions and experiences as this will help us make recommendations that are feasible and will help meet the needs of these populations. Your refusal to participate will not affect their job or employment.

Do you have any questions before we start? *(Take time to address all questions and concerns)*

1. GENERAL INFORMATION/TRENDS

Let's start by talking about the MSM population in [name of the survey area].

1.1. How would you describe in general the population of MSM in [name of the survey area]?

1.2. Have you noticed any changes or trends over the past year with regard to MSM? (e.g. new populations/groups, new hangouts, new or changing risk behaviors)

2. BACKGROUND QUESTIONS ABOUT THEIR ORGANIZATION

The following questions relate to your organization and their interaction with MSM.

2.1. What is your role at your clinic/organization?

2.2. What kinds of services does your organization provide?

2.3. How have you come to know MSM?

2.4. If yes, in what context do you interact with them?

2.5. What kinds of services does your organization provide?

2.6. What have been some of the challenges for you or your organization in providing services to or interacting with MSM?

2.7. What have been some of the successes? What has worked well?

2.8. What do you think should be the priorities for providing services to MSM?

2.9. When you come into contact with MSM when you are working with them, how do you know they are MSM?
   a. *What are your feelings about MSM in general?*

2.10. What kinds of health problems do MSM present with?
   a. *Are there problems such as anal warts, lesions that are specifically related to their sexuality?*
   b. *How to they explain that they have such problems (directly or indirectly)? Please give a specific example.*
   c. *Could you describe a time when mental health is discussed?*

2.11. Can you describe how you interact with MSM as part of the work in your clinic/ organization?
   a. *Can you discuss their problems openly with them?*
   b. *What techniques do you use for getting them to talk openly? Please give a specific example.*

2.12. **Only for those that offer psycho-social support**: What type of psycho-social support do you offer to MSM?
   a. *Probe to see if s/he has ever treated anyone for problems associated with violence, for example rape or beating.*
   b. *What are the psycho-social needs of MSM?*
   c. *How do think these psycho-social needs can be met?*

2.13. What are the problems MSM experience when they consult a health practitioner?
   a. *Probe to see if there is stigmatization, perception of lack of confidentiality, etc.*
   b. *How can their experiences be improved? (Probe for characteristics of health practitioners, any particular services, separate facilities, etc)*

2.14. Among your colleagues, what proportion is comfortable working with MSM?
   a. *How do health care providers ensure confidentiality?*

3. ACCEPTABILITY OF SURVEY AND MSM PARTICIPATION

Say to participant:

As you know, we are planning a future survey of MSMs. We will ask MSM to recruit each other for the survey. In the future survey we will interview MSMs about HIV and what they do to prevent it. We will ask questions about how they meet people and about things they do that might put them at risk for HIV. We also want to give free HIV and STI testing and counselling. People who choose to be tested for HIV and STIs will learn their test results. If they test positive for HIV, we will refer them for care and treatment. The future survey will take about two hours for participation.

3.1. What are your initial thoughts about this survey?
   a. *How would MSMs feel about participating in this survey?*
   b. *What would discourage them from participating in this survey?*
   c. *What would make them feel more comfortable about participating?*

3.2. Only for clinicians: We plan to offer free testing and treatment for HIV and sexually transmitted infections as part of the survey. The tests involve collecting some blood. What challenges have you experienced collecting blood from people in general and specifically from MSMs?

Probe for fear of learning they have HIV, fear of lack of confidentiality, infecting others, use in “witchcraft”, etc.
   a. *What would make participants feel more comfortable providing specimens?*
   b. *Would people feel more comfortable with a male or female nurse? Any other things?*

3.3. We will offer HIV and STI testing and referral. What other services would MSMs benefit from in your view?

Probe to see what services are currently lacking or how existing services could be improved (medical, psychosocial, etc.)

3.4. We want this survey to be helpful to MSMs We also want to make it safe for people to join. What can we do to keep people safe?

3.5. Should we tell the police or other law enforcement agencies about the survey so they don't bother people trying to join? Or is it better not to inform them? What about others (religious, military, local level government, etc.)?

3.6. How do you think more hidden MSMs will feel about joining the survey?

a. What can we do to make it easier for such MSMs to join the survey?

b. Are there any groups or individuals we can mobilize to make it easier for more hidden MSMs to participate?

3.7. What type of people do you know who are influential among MSMs? Peer leaders? Gatekeepers? This person need not be MSMs. This would be someone who knows a lot of other MSMs and is well liked by peers. Would they be willing to talk to us?

4. LOCATION, DAYS, TIMES

4.1 What kind of place would you like to see as the main survey site location in this area? *What might be some other convenient and safe locations?*

4.2 Where would you and your peers feel comfortable coming to participate in a survey? Rented apartment or office? Clinic? NGO? Other location?

4.3 How easy is it for people to get there?

4.4 How do you think you and your peers would travel to the survey site?

a. Probe for public transport, own vehicle, shared taxi, private taxi, etc.

4.5 About how much would you expect transport to the main interview site will cost for most people?

a. Probe to see if this cost is for public transit, shared taxi, private taxi, private motorcycle etc.

4.6 What areas of town are unsafe and should be avoided for the survey?

4.7 What types of MSMs would not feel comfortable going to certain areas?

4.8 Are there any locations where you would not want to be surveyed?

4.9 What would make a survey site most comfortable for other MSMs?

4.10 What times of the day are best for MSMs to go to an interview site to take part in a survey?

a. Morning (8 AM–12 PM)?

b. Afternoon (12 PM–5 PM)?

c. Evening (5 PM–10 PM)?

4.11 What days of the week do you think MSMs are best for people to join the survey? *What about the weekend?*

4.12 Would appointments or open-walk in times work better?

4.13 We may also ask other MSMs to participate in a similar study at the same time. How would you feel about being at the same survey site as they?

That is the end of our interview.

Thank you so much for sharing your thoughts with me.

Do you have any questions, or is there anything that you would like to add before we end?

If you have further thoughts about any of the issues we discussed today, please call *[INSERT NUMBER WHERE INTERVIEWER CAN BE CONTACTED]*

TO BE COMPLETED BY THE INTERVIEWER:

Please note your impressions about the session, its main themes and the comments and reactions of participants

INTERVIEW WAS: ____ ROUTINE ____ NOT ROUTINE

IF NOT ROUTINE, WHY:

ANY ADVERSE REACTIONS IN THE INTERVIEW: ____ YES ____ NO

IF YES, SPECIFY:

OTHER OBSERVATIONS/COMMENTS:
