# Supplementary material for: Characterizing acceptable and appropriate implementation strategies of a biobehavioral survey among men who have sex with men and others assigned male who have sex with men in Zimbabwe
Source: PLOS Glob Public Health. 2022 Oct 26;2(10):e0001097. doi: 10.1371/journal.pgph.0001097 (PMC10021218; doi:10.1371/journal.pgph.0001097)
Supplement: Attachment — Submitted filename: Response to Reviewers 17 Aug 2022.docx [file pgph.0001097.s006.docx]

Dear Editors,

Thank you to the reviewers for the constructive feedback on the manuscript, and for the opportunity to revise and resubmit. We have addressed point by point each reviewer comment, improving the manuscript to address reviewer concerns. Moreover, if necessary, we are happy to rapidly address further concerns. We believe that formative work among men who have sex with men and others assigned male who have sex with men (MSM/OAMSM) remain an important and under published area necessary to inform public health surveillance efforts and broader HIV-related engagement efforts among MSM/OAMSM in Zimbabwe and other contextually similar contexts.

***Reviewer 1:***

***Thank you for the opportunity to review the manuscript
The paper is well written, and addresses gaps in literature relating to key populations’ behaviour survey which are important in HIV related engagements and interventions for this group.
The data and data analysis do support the claims; however, the exclusion criteria have not been sufficiently explained***
Response: Inclusion/exclusion criteria are described in Lines 98-101 and 107-113 for MSM/OAMSM and key informant participants, respectively, and additional information has been provided. Any individuals meeting the inclusion criteria were eligible to participate in the formative assessment. ***In light of the fact that same sex relationships are criminalised in Zimbabwe, it is understandable that the informed consent were given verbal. However, the authors need to provide the ethical clearance number.***
Response: We have updated the text and provided the ethical clearance numbers in Lines 165-168. ***The paper is well written and is suitable for publication***

***Reviewer 2: SUMMARY AND OVERALL COMMENTS

This manuscript describes the formative research undertaken prior to the implementation of a respondent driven sampling (RDS) bio-behavioural survey (BBS) among GBMSM in Harare and Bulawayo in Zimbabwe, where a previous BBS among MSM had not until that point been published in a peer-reviewed journal. The approach taken was sound and the findings consistent with the small amount of previous research and programmatic work in Zimbabwe and in other settings sharing some similar contextual characteristics. The authors make clear how their findings informed survey design. As the authors mention, the findings from similar qualitative formative work conducted to inform RDS survey design are often not published or reported in much detail, so it is a helpful contribution to the literature and to the implementation of future research to do so. The paper is nicely written and well-organised.

There were a few questions I had remaining after reading the article:
- were the researchers explicitly aiming to recruit cisgender MSM only? Please clarify and if not, please clarify the extent to which participants included diverse gender identities. There is mention of this in the discussion but I think it needs to be clear what the intended population to recruit was earlier on and I still easn't clear what the original intentions of the survey were.***
Response: As noted in the discussion, based on stakeholder feedback received after the formative assessment, transgender women were actively recruited to participate in the BBS as purposively selected seeds. As eligibility criteria was specific to male sex assigned at birth for both the formative assessment and biobehavioral survey, individuals who met eligibility criteria were able to participate regardless of gender identity. In the formative assessment, participants were screened for eligibility but were not specifically asked about their gender identity (as noted in the limitations). We have clarified in the methods that *“While cis-gender MSM were the original target population for the formative assessment, eligibility criteria were specific to male sex assigned at birth (for both the formative assessment and subsequent biobehavioral survey), and individuals were eligible to participate irrespective of gender identity.”* We have also revised the language throughout to clarify participants in the formative assessment represent MSM and others assigned male who have sex with men in response to this comment.

***- to what extent were the FGD and IDI participants (not key informants) representative of MSM who were not engaged with MSM community groups/service providers/prior research already?***
Response: Given the cultural and legal context in Zimbabwe, it was imperative that we work closely with trusted entities in the MSM/OAMSM community and engaged several community-based organizations working with lesbian, gay, bisexual, transgender, and intersex (LGBTI) populations, including GALZ, TranSmart, PSI, Pangaea, CESHHAR, Hands of Hope, and Sexual Rights Centre, to support recruitment. As noted, we also recruited participants through snowball sampling. Qualitative research is not intended to be generalizable given contextual nuances and an inherent limitation of our recruitment approach is that participants may already have contact with key population (KP)-friendly services, self-identify as “gay”, or perceive that they are at less risk for harms related to being MSM/OAMSM than those who did not participate. As such, the formative assessment may not represent all perspectives shared by individuals in the MSM/OAMSM community in Harare and Bulawayo. This is noted as a limitation in the discussion.

***- what did the authors find regarding the existence of sub-communities likely present within the social network and the characteristics by which they varied?***
Response: Participants did describe some sub-communities including male sex workers who sell sex, MSM/OAMSM who have sex with/date/marry women, and differences in MSM/OAMSM sub-communities by ethnic, socioeconomic, and age groups. It was reported by a couple of participants that white MSM/OAMSM were less engaged in KP community groups. Younger MSM/OAMSM <18 years of age were also reported not to engage in KP community groups as they were considered underage. However, we did not feel we reached saturation on some of these themes and have omitted them from the results. As indicated in our original submission, participants reported that younger MSM/OAMSM were more likely to openly self-identify as gay or homosexual, while older MSM/OAMSM were more discrete about their sexual orientation and behaviors due to ostracization in the past and/or fear of losing one’s social standing. Participants reported that older MSM/OAMSM often had wives or live-in girlfriends, including lesbian women, to conceal their sexual orientation, and were less likely to engage with KP-friendly organizations compared to younger MSM/OAMSM. As we feel this theme reached saturation, we have added additional information on MSM/OAMSM engaging in romantic relationships with women to the results.

***A key point was that I was curious about was whether, on reflection, there were any topics NOT covered by the formative research that the researchers later realised would have been useful to cover. I think the main value of this article is in providing a worked example of doing such formative research prior to implementation of a a similar survey in a similar population, so it would be useful to know this***
Response: As noted in the limitations section, our formative assessment tools did not capture information specific to gender identity which limited our ability to explore acceptability and appropriateness of BBS approaches for transgender women and/or non-binary or gender fluid individuals. Another area was related to economic context in Zimbabwe. A major operational challenge for the BBS was Zimbabwe’s severe hyper-inflation, which drastically worsened during survey data collection. Additionally, toward the end of the BBS, Zimbabwe implemented a water rationing program due to acute water shortages, limiting water to once per week for some residents, and rationing of electricity with power cuts of up to 18 hours per day. While the fluctuating socioeconomic situation was explored to some degree during the formative assessment as part of compensation-related probes, formative assessment tools did not explore considerations of instability in-depth, limiting our ability to fully anticipate implementation strategies in the context of an uncertain operating environment. We have added text on this to the paper. ***I have some comments, including minor typos/edits suggested for each section below.

ABSTRACT:
Suggest re-phrasing to avoid repeated word: “Yet the marginalized nature of populations participating in a BBS requires contextually acceptable and appropriate implementation strategies for effective implementation.”***
Response: We have removed the first use of ‘implementation’ from this sentence. ***INTRODUCTION
Line 63: suggest ‘about’ MSM instead of ‘on MSM’.***
Response: This has been updated. ***METHODS:
Were there any eligible potential participants who declined to take part in the study?***
Response: No, all potential participants who were screened for eligibility and found to be eligible took part in the study. We have updated the results section to respond to this comment. ***Holding focus groups separately for older and younger MSM makes sense to me. Were there any other key aspects that researchers might have considered important such as sexual orientation, gender identity or known HIV status that researchers might have considered separate groups for?***
Response: Thank you for this comment. Sexual orientation, gender identity and known HIV status are all potentially important characteristics to stratify focus groups by. In some ways we were constrained by resources in that we conducted only two focus groups per city and in consultation with the LGBTI groups we worked with age was prioritized over other stratifications. Certainly, as other researchers have pointed out, transgender women have unique needs and distinct challenges when accessing HIV services compared to MSM (https://www.ncbi.nlm.nih.gov/pmc/articles/PMC7836207/). As noted in the limitations section, gender identity was not explicitly explored in nor a stratification of focus groups in this formative assessment.

***Were any other survey designs other than RDS explicitly discussed with FGD and IDI participants, eg venue-based methods? I’m not suggesting that this necessarily would have been more appropriate but I was interest interested in the breadth of what was considered and discussed and reasons why other survey designs might not have been appropriate.***
Response: We did include a probe within the guide*, “For the future HIV survey, would you like us to come to the places you socialize to do the survey”* however this optional probe was not used consistently across focus groups and the depth of results centered around operationalizing RDS. Given participant feedback that many MSM/OAMSM were closeted, venue-based methods would likely produce a biased sample and not be generalizable to the target population. Per your comment below, we have attached the guides as supplementary materials.

***In the spirit of providing a useful resource to other future projects, it would be helpful to attached topic guides and/or other data collection instruments used for this study as an appendix.***
Response: We appreciate this feedback and have attached the data collection instruments as supplemental materials. ***RESULTS
Line 169: Here do you mean that the individual interviewees had been involved in service provision for a median of 2 years or that the organisations had only existed this long?***
Response: Two years is the median number of years key informant participants had provided services to MSM/OAMSM. We have revised the language to ensure this is clear, *“Key informant participants who provided healthcare services to MSM/OAMSM had provided services for a median of 2 years and saw a median of 10 MSM/OAMSM patients per week.”* ***I would find it helpful to better understand the extent to which interviewees and FGD participants were already in touch with LGBT community groups, or service provision. I understand that not all participants were identified by service providers and that snowball sampling was used, but am still curious about how representative the participants might have been of MSM not already served by community groups and clinics. I wondered about this particularly as it was reported Line 202 that many MSM participants had prior experience with HIV testing in research and clinical settings (though I suppose the latter would surprise me less). Also Line 333, where it is implied that some some number had previously been compensated in the context of public health programmes. I see that the authors have commented on this in the Discussion , which is good, but was there any information about this collected from participants that could inform this question?***
Response: We did not collect any additional information which could inform this question. Given the cultural and legal context in Zimbabwe, it was imperative that we work closely with trusted entities in the MSM/OAMSM community and engaged several community-based organizations working with LGBTI, including GALZ, TranSmart, PSI, Pangaea, CESHHAR, Hands of Hope, and Sexual Rights Centre, to support recruitment. As noted, we also recruited participants through snowball sampling. Qualitative research is not intended to be generalizable given contextual nuances and an inherent limitation of our recruitment approach is that participants may already have contact with KP-friendly services, self-identify as “gay”, or perceive that they are at less risk for harms related to being MSM/OAMSM than those who did not participate. As such, the formative assessment may not represent all perspectives shared by individuals in the MSM/OAMSM community in Harare and Bulawayo. This is noted as a limitation in the discussion.

***On compensation, was any separate data collected on compensation in the context of onwards recruitment for the RDS surveys (as these surveys typically compensate participants for both their own participation and for onwards recruitment)?***
Response: We did include a probe within focus group guides related to compensation for peer recruitment, *“We would like to compensate participants who recruit peers. What do you think we should give participants for recruiting peers?"* This optional probe was not used in all focus groups, so we did not reach saturation on this theme.

***Were there any differences reported in the extent to which younger and older MSM might be well networked or not?***
Response: When describing challenges in terms of recruiting older MSM/OAMSM, participants emphasized older MSM/OAMSM’s lack of openness in sexual orientation, not specifically the size of their networks. Participants reported that younger MSM/OAMSM were more likely to openly self-identify as gay or homosexual, while older MSM/OAMSM were more discrete about their sexual orientation and behaviors due to ostracization in the past and/or fear of losing one’s social standing. While it is possible that the tendency of older MSM/OAMSM to be less open about sexual orientation relates to network size, this was not specifically mentioned in the data, and we therefore did not infer a connection.

***Was there any information collected about sub-communities that might be expressed as sub-networks for the RDS recruitment, and if so, what was found?***
Response: Participants did describe some sub-communities including male sex workers who sell sex, MSM/OAMSM who have sex with/date/marry women, and differences in MSM/OAMSM sub-communities by ethnic, socioeconomic, and age groups. It was reported by a couple of participants that white MSM/OAMSM were less engaged in KP community groups. Younger MSM/OAMSM <18 years of age were also reported not to engage in KP community groups as they were considered underage. However, we did not feel we reached saturation on some of these themes and have omitted them from the results. As indicated in our original submission, participants reported that younger MSM/OAMSM were more likely to openly self-identify as gay or homosexual, while older MSM/OAMSM were more discrete about their sexual orientation and behaviors due to ostracization in the past and/or fear of losing one’s social standing. Participants reported that older MSM/OAMSM often had wives or live-in girlfriends, including lesbian women, to conceal their sexual orientation, and were less likely to engage with KP-friendly organizations compared to younger MSM/OAMSM. As we feel this theme reached saturation, we have added additional information on MSM/OAMSM engaging in romantic relationships with women to the results.

***Application to survey methodology- this section was reported quite positively and it does indeed sound like the formative research was useful and important to have done. I was curious though whether there were any topics that in hindsight the researchers wish they had had more data to inform that they could have collected at the formative stage?***
Response: As noted in the limitations section, formative assessment tools did not capture information specific to gender identity which limited our ability to explore acceptability and appropriateness of BBS approaches for transgender women and/or non-binary or gender fluid individuals. A major operational challenge for the BBS was Zimbabwe’s severe hyper-inflation, which drastically worsened during survey data collection. Additionally, toward the end of the BBS, Zimbabwe implemented a water rationing program due to acute water shortages, limiting water to once per week for some residents, and rationing of electricity with power cuts of up to 18 hours per day. While the fluctuating socioeconomic situation was explored to some degree during the formative assessment as part of compensation-related probes, formative assessment tools did not explore considerations of instability in-depth, limiting our ability to fully anticipate implementation strategies in the context of an uncertain operating environment. We have added text on this within the manuscript.

***DISCUSSION

The research conducted (even if unsuccessfully) among MSM in the Caribbean needs a reference of some kind.***
Response: This reference was cited in the previous sentence. We have updated the subsequent sentences to ensure the reference is also provided.

***Line 450: It wasn’t clear to me from what was said why issues specific to transgender women were not captured when they were included in the IBBS. Was their inclusion recommended only after completion of the formative research? It might also be helpful to clarify the gender identity of participants and the gender criteria for the final study earlier on because I was wondering about this while reading the paper and it is not mentioned until the discussion.***
Response: As eligibility criteria was specific to male sex assigned at birth for both the formative assessment and biobehavioral survey, individuals who met eligibility criteria were able to participate regardless of gender identity. In the formative assessment, participants were screened for eligibility but were not specifically asked about their gender identity (as noted in the limitations). We have clarified in the methods that *“While cis-gender MSM were the original target population for the formative assessment, eligibility criteria were specific to male sex assigned at birth (for both the formative assessment and subsequent biobehavioral survey), and individuals were eligible to participate irrespective of gender identity.”* We have also revised the language throughout to clarify participants in the formative assessment represent MSM and others assigned male who have sex with men.
